# Editorial: Addressing emotionally based school avoidance: causes, consequences, and interventions

**DOI:** 10.3389/frcha.2026.1802875

**Published:** 2026-03-25

**Authors:** Joanna K. Anderson, Trude Havik, Carolina Gonzálvez

**Affiliations:** 1 University of Cambridge, Cambridge, United Kingdom; 2 Universitetet I Stavanger, Stavanger, Norway; 3 Universitat D'Alacant Facultat D'Educacio, Alicante, Spain

**Keywords:** bioecological model, emotionally based school avoidance (EBSA), mental health, school absence, school attendance problems

School attendance problems (SAPs) are a rapidly growing concern across many countries, with rates rising further in the aftermath of the COVID-19 pandemic (1, 2). SAPs are not a unitary construct but encompass multiple forms, each shaped by interacting factors across individual, relational, organisational, and societal levels (3). Emotionally based school avoidance (EBSA) refers to severe and persistent emotional distress associated with attending school, resulting in significant avoidance and prolonged absence (4). EBSA is one of the most complex forms of SAPs and is rapidly rising in prevalence, typically associated with co-occurring mental health needs and school-related stressors, with the school environment factors often acting as triggering conditions.

High levels of school absence, regardless of underlying causes, are associated with a broad range of negative outcomes. These include low academic attainment, school dropout, impaired social functioning, and, in the longer term, reduced employment prospects, financial instability, poorer physical and mental health, and higher risk of engaging in antisocial or delinquent behaviours (5–7). Vulnerable children and young people are disproportionately affected, exacerbating existing inequities.

This Research Topic aims to disseminate novel research and emerging theoretical frameworks that advance our understanding of the complex drivers of EBSA and inform practical, evidence-based responses and interventions. In addition, it explores how EBSA relates to other forms of SAPs, identifying overlaps and distinctions, and their implications for prevention and intervention efforts.

Across contributions, a consistent message emerges: EBSA and SAPs are driven by multiple interacting factors rather than a single, distinct cause. They are shaped by multi-level, dynamic processes in which attendance emerges from interacting influences across individual, relational, institutional, societal, and temporal levels. Therefore, bioecological frameworks are particularly relevant, as they conceptualise development as the product of ongoing interactions between individuals and the systems in which they are embedded (8).

While the papers in this special issue address influences across multiple system levels, the largest number focus on individual-level factors, particularly emotional distress and cognitive–motivational processes. Multiple studies identify internalising symptoms, particularly anxiety and depression, as primary drivers. Using network analysis, Alanko et al. demonstrate that depression and social anxiety occupy central positions within symptom and function networks of SAPs. Krause et al. present longitudinal evidence from Canada showing a bidirectional relationship between mental health difficulties and poor attendance, while trajectory modelling by Brittain and Vaillancourt indicates that mental health and attendance are “co-developing intertwined trajectories.” Several studies highlight the role of internal psychological mechanisms. Perez-Marco et al. show that learned helplessness—a pattern in which students believe they cannot succeed academically—has strong predictive and discriminative value in identifying students at high risk of SAPs. Similarly, Sorrenti et al. show an association between students’ lower awareness and acceptance of present thoughts, feelings, and social experiences (mindfulness) and increased peer and emotional difficulties, which contribute to school refusal. Sasso reports that for autistic students, absenteeism often functions as their coping response to sensory overload, exhaustion and social interaction.

Research focusing on microsystem-level factors emphasises the role of relationships. Trusting relationships are identified as key factors supporting regular attendance. Ekornes et al. highlight that teachers identify students of concern by assessing deviations from the individual child's ‘typical’ functioning rather than relying solely on general age norms, emphasizing the value of close teacher-student knowledge. Hamadi et al. highlight how difficulties in the student-teacher relationship and delayed or mismatched professional responses can drive feelings of alienation and SAPs. Parent- focused studies by Hansen et al. and Knollmann and Reissner underscore the importance of respectful relationships between parents and professionals; interventions succeed when parents feel empowered and are included in decision- making.

Furthermore, a systematic review by Alaimo et al. underscores that school staff's conceptualisation of non-attendance varies widely, distinguishing among “within-child”, “home”, and “school-related” factors that directly shape the support strategies they employ. Enderle et al. demonstrate that while professionals often focus on procedures, students prioritise emotional safety and being understood. Practice-focused studies demonstrate how relational approaches support attendance. Alaimo and Kelly demonstrate how participatory, school-owned relational models support attendance, while Karel et al. reports that outcomes of the @School intervention, a modular, developmentally sensitive Cognitive Behavioral Therapy (CBT) program designed to support neurodiverse adolescents with emotion-related school attendance challenges, improve when families and schools collaborate around shared attendance goals. Bania et al. evaluation of the pilot of Back2School intervention, also a modular CBT that draws on core components from the @School program, developed to address a broader range of problematic school absenteeism, indicates that teachers are vital partners, and their involvement needs to be clearly defined.

At the exosystem level, research highlights the role of school-level structures. Heyne et al. draw attention to the often-overlooked category of authorised absence. Their action research in the Netherlands reveals that schools exert significant influence over authorised leave, suggesting that addressing school-initiated absences is critical.

School belonging emerges as a key protective factor in multiple studies, including Whitley et al., and trajectory research by Brittain and Vaillancourt, while transition points are consistently identified as risk accelerators. Whitley et al. further identified unmet support needs and school-initiated exclusion as systemic factors that undermine inclusive education. Transition points, particularly entry into secondary school, are consistently identified as risk accelerators in longitudinal modelling.

Systematic review evidence from Middleton et al. shows mixed but promising effects of school-based interventions, including mentoring, counselling, family-linked approaches, and school-health integrated approaches. Neumann et al. address the critical gap between clinical care and education by presenting the protocol for “SchuTIng-stAR”, a seamless stepwise rehabilitation program designed to support sustainable reintegration following psychiatric inpatient treatment.

At the macrosystem level, the special issue underscores that SAPs are fundamentally equity issues. Whitley et al. document disproportionately high levels of absence among students with disabilities and those from lower-income households. Complementing this, Chen uses computational text analysis to demonstrate how national and societal expectations regarding academic performance in China shape attitudes, pressures, and institutional practices that influence EBSA. Comparative contributions across Europe, North America and Asia further illustrate how policy frameworks shape attendance responses. A key conceptual contribution by Kearney reframes chronic absenteeism as a public health issue, calling for cross-sector responses.

Studies framed within the chronosystem highlight that SAPs are developmental outcomes that are progressive and cumulative. Hamadi et al. describe a gradual onset of SAPs rather than a sudden withdrawal. Developmental trajectory work by Brittain and Vaillancourt further shows co-evolving patterns of attendance and academic achievement, with school transitions emerging as periods of elevated risk. In addition, Krause et al. analyses of longitudinal data during the COVID-19 pandemic highlight shifting patterns of absence.

The articles in this special issue demonstrate that SAPs and EBSR are complex, developmentally embedded phenomena. Effective responses cannot be achieved through isolated or child-focused interventions alone. The findings point to the need for integrated, multi-tiered approaches that align supports across education, mental health, and social care systems (9, 10). Schools play a pivotal dual role as sites of both risk and protection and therefore require sustained support to implement equitable, preventative, and data-informed attendance practices. Collectively, the contributions in this Research Topic call for a paradigm shift in understanding emotionally based school avoidance. Future progress depends on cross-sector collaboration, early and developmentally informed prevention, and policies that enable flexible and inclusive school responses.
